# Unraveling cutaneous histiocytosis: insights into histology, pathogenesis, diagnosis, and treatment pitfalls

**DOI:** 10.3389/fmed.2025.1585815

**Published:** 2025-06-20

**Authors:** Lucian G. Scurtu, Francesca Scurtu, Olga Simionescu

**Affiliations:** ^1^Faculty of Medicine, “Carol Davila” University of Medicine and Pharmacy, Bucharest, Romania; ^2^Department of Dermatology I, Colentina Clinical Hospital, Bucharest, Romania; ^3^Department of Obstetrics and Gynecology, Filantropia Clinical Hospital, Bucharest, Romania

**Keywords:** histiocytosis, langerhans cell histiocytosis, mononuclear phagocyte system, CD1a, CD207, langerin, mitogen-activated protein kinase, BRAF inhibitor

## Abstract

Histiocytoses represent a group of diverse rare disorders characterized by the abnormal accumulation of cells derived from the mononuclear phagocyte system in various tissues and organs. The mononuclear phagocyte system includes monocytes, macrophages, dendritic cells, and specialized tissue-resident phagocytes. These cells are essential for both innate and adaptive immunity and preserving tissue homeostasis. Several classifications of histiocytoses by the Histiocyte Society (1987, 1997, 2016) and WHO (2018, 2022) and an International Consensus Classification (2022) are generally acknowledged. The WHO 2022 classification clarifies these heterogeneous disorders by dividing them into three major groups. Cutaneous involvement in histiocytosis is often polymorphous, making clinical decision more challenging. Cutaneous histiocytoses can occur either as primary cases or as a manifestation of a multisystemic disease. In support of the standard pathology report, immunohistochemical staining is warranted. The exact etiopathogenesis of histiocytoses remains poorly understood, and various associations with malignancies, including visceral and hematologic cancers, as well as autoimmune diseases and infections (Borrelia burgdorferi) are still under review. One of the most recent advancements in this field is the discovery of somatic mutations in the RAF-MEK-ERK signaling pathway, particularly BRAF mutations. Oncogene-induced senescence-associated BRAF mutations have been described in Langerhans cell histiocytosis and Erdheim-Chester disease. Targeted therapies with BRAF inhibitors such as dabrafenib and vemurafenib have shown promising results. MEK inhibitors, like trametinib and cobimetinib, have demonstrated efficiency regardless of the BRAF mutation status. Local treatments of cutaneous histiocytosis include topical steroids, calcineurin inhibitors, alkylating agents, phototherapy, steroid injections, and laser therapies. Despite the current advances in pathogenesis and treatments, cutaneous histiocytosis stands as a challenging and heterogeneous group of disorders, and treatment guidelines are warranted.

## 1 Introduction

Histiocytoses are a group of rare disorders produced by the accumulation of cells derived from monocytes and macrophage lineages in different tissues and organs. These conditions can affect both pediatric and adult populations. Their clinical manifestations vary widely, ranging from cutaneous localized forms to more severe, multisystem involvement. The progression of the disease can be indolent, with some cases resolving spontaneously, while others may follow a more aggressive course (1, 2).

The first classification of histiocytosis was published in 1987 by the Histiocyte Society and comprised three categories: Langerhans, non-Langerhans, and malignant histiocytoses. The initial classification was refined in 1997, and included disorders of varied biological behavior and malignant lesions (3). In 2016, the Histiocyte Society introduced a revised and improved classification of histiocytosis (Table 1), which, in contrast to the 1987 classification, incorporated a comprehensive approach that included histological features, phenotypic characteristics, molecular alterations, and clinical and imaging findings. Thus, 5 groups of histiocytoses have been described: Langerhans-related (L Group), cutaneous and mucocutaneous (C Group), malignant histiocytoses (M Group), Rosai-Dorfman disease (R group), and hemophagocytic lymphohistiocytosis and macrophage activation syndrome (H Group) (4). In 2018, the World Health Organization (WHO) classification of skin tumors (5) presented a new classification and updated it 4 years later, in the 5th edition of the WHO classification (2022), where a more nuanced approach was taken by categorizing dendritic cells and histiocytic neoplasms after myeloid neoplasms, reflecting their common origin from myeloid progenitors (Table 2). This updated system divides these neoplasms into three categories:

1. Plasmacytoid dendritic cell neoplasms: this group includes both mature forms associated with myeloid neoplasms and their blastic variants.
2. Langerhans cell and other dendritic cell neoplasms: this category encompasses conditions such as Langerhans cell histiocytosis (LCH) and related sarcomas. It also includes tumors arising from indeterminate and interdigitated cells.
3. Histiocytic neoplasms: this category includes a variety of disorders such as juvenile xanthogranuloma (JXG), Erdheim-Chester disease (ECD), Rosai-Dorfman disease (RDD), ALK-positive histiocytosis, and histiocytic sarcoma (6).

**TABLE 1:** Histiocyte Society classification of histiocytoses **(2016)**.

Histiocytoses	
L Group	– LCH – ICH – ECD – Mixed LCH/ECD
C Group	– Cutaneous non-LCH: • Non-XG family: cutaneous RDD, NXG, cutaneous
	histiocytoses not otherwise specified • XG family: JXG, AXG, SRH, BCH, GEH, PNH – Cutaneous non-LCH with a major systemic component: • XG family: • XD Non-XG family: MRH
R Group	– Familial Rosai-Dorfman Disease (RDD) – Sporadic RDD: classical, extra-nodal, RDD with neoplasia or immune disease, unclassified
M Group	– Primary Malignant Histiocytoses – Secondary Malignant Histiocytoses
H Group	– Primary HLH: Monogenic inherited conditions leading to HLH – Secondary HLH (non-Mendelian HLH) – HLH of unknown/uncertain origin

LCH, Langerhans cell histiocytosis; ECD, Erdheim-Chester disease; ICH, indeterminate cell histiocytosis; XG, xanthogranuloma; XD, xanthoma disseminatum; RDD, Rosai-Dorfman disease; NXG, necrobiotic xanthogranuloma; JXG, juvenile xanthogranuloma; AXG, adult xanthogranuloma; SRH, solitary reticulohistiocytoma; BCH, benign cephalic histiocytosis; GEH, generalized eruptive histiocytosis; MRH, multicentric reticulohistiocytosis; HLH, hemophagocytic lymphohistiocytosis.

**TABLE 2:** The 5th edition of the WHO classification of dendritic cell and histiocytic neoplasms.

**1. Plasmacytoid dendritic cell neoplasms:** • Mature plasmacytoid dendritic cell proliferation associated with myeloid neoplasm • Blastic plasmacytoid dendritic cell neoplasm
**2. Langerhans cell and other dendritic cell neoplasms:** – Langerhans cells neoplasms: • Langerhans cell histiocytosis • Langerhans cell sarcoma – Other dendritic cell neoplasms: • Indeterminate dendritic cell tumor • Interdigitating dendritic cell sarcoma
**3. Histiocytic neoplasms:** • Juvenile xanthogranuloma • Erdheim-Chester disease • Rosai-Dorfman disease • ALK-positive histiocytosis • Histiocytic sarcoma

In the International Consensus Classification (ICC) of Mature Lymphoid Neoplasms published in 2022 (7), RDD and ALK-positive histiocytosis were regarded as distinct entities and gained autonomous recognition.

Cutaneous involvement in histiocytosis often presents with polymorphous lesions, making clinical diagnosis challenging (8). This review aims to present the key histopathologic and pathogenic features of cutaneous histiocytosis in light of recent molecular findings and innovative therapies.

## 2 The mononuclear phagocyte system

The mononuclear phagocyte system (MPS), ex-reticuloendothelial system, is an essential component of the immune response. It comprises a diverse network of cells critical for both innate and adaptive immunity and for maintaining tissue homeostasis through removing dead cells and waste products. The MPS includes monocytes, macrophages, dendritic cells, and specialized tissue-resident phagocytes (9, 10).

As the largest white blood cells, monocytes account for about 5–10% of the circulating nucleated cells and play a crucial role in patrolling for microorganisms and orchestrating immune responses during infections. Monocytes possess toll-like receptors (TLRs) that recognize pathogen-associated molecular patterns (PAMPs) on invading microbes, prompting them to migrate from the bone marrow into the bloodstream and infiltrate tissues within 24–48 h (11–14). Under steady-state conditions, monocytes represent a diverse population of cells that circulate between the blood, spleen, and bone marrow. They are rapidly recruited to sites of inflammation and can give rise to a variety of cell types. In the skin, these include dermal macrophages, dermal dendritic cells, and epidermal Langerhans cells (15).

In 1868, the student Paul Langerhans first identified Langerhans cells (LCs). These cells were located in the cutaneous epidermis and exhibited a morphology reminiscent of neurons due to their dendritic structures (16). Langerhans cells account for approximately 3–5% of all nucleated epidermal cells (17). LCs extend their dendrites through tight junctions toward the stratum corneum, enabling them to survey for antigens across multiple layers of the epidermis. They are capable of capturing and processing foreign antigens. The maintenance of LCs in homeostatic conditions within the epidermis is achieved through self-renewal, which offsets the constant low-level migration of these cells from the epidermis to the draining lymph nodes. By their developmental origin, LCs are appropriately classified within the macrophage lineage. Genetic analyses indicate that LCs are distinctly differentiated from macrophages and conventional dendritic cells (DCs) during steady-state conditions (18–22).

DCs originate from a lineage derived from hematopoietic stem cells (HSCs) in the bone marrow, dependent on the receptor tyrosine kinase FLT3. DCs reside in the cutaneous epidermis and dermis. Patients exhibiting deficiencies in blood monocytes and DCs due to mutations in IRF8 and GATA2 show a lack of specific dermal DC subsets and a reduced number of macrophages, while the population of LCs remains intact. This observation suggests that dermal DCs are directly dependent on circulating monocytes and/or DCs, or may arise from a shared precursor derived from hematopoietic stem cells (20). DCs serve as professional antigen-presenting cells and capture antigens through specialized surface receptors. In contrast to myeloid DCs, plasmacytoid DCs (pDCs) are practically undetectable in healthy skin but are recruited during inflammation (23, 24).

Metchnikov first characterized macrophages as phagocytes, initiating over a century of research into tissue-resident macrophages located at barrier surfaces like the skin. They are not just passive scavengers but actively contribute to homeostatic functions (25). Macrophages display two important phenotypes in response to microenvironmental signals, which give rise to M1 and M2 populations, a process known as macrophage polarization. M1 macrophages are known for producing pro-inflammatory cytokines and displaying strong microbicidal abilities. On the other hand, M2 macrophages act as anti-inflammatory cells, resolve inflammation, and promote wound recovery *via* angiogenesis (26, 27).

## 3 Histopathology advancements in cutaneous histiocytoses

### 3.1 LCH, ICH

An infiltrate of abnormal MPS cells characterizes histiocytoses. In LCH, the cells are large, round to oval, with a pale cytoplasm, and reniform nuclei with clustered, subepidermal arrangement. Atypical mitosis and marked pleiomorphism are unusual, and if present, should raise suspicion of Langerhans cell sarcoma. LCH cells lack branching and are accompanied by an inflammatory infiltrate consisting of eosinophils (eosinophilic granuloma pattern), neutrophils, lymphocytes (lichenoid dermatitis pattern), and macrophages. Eosinophils are frequent and usually scattered through the infiltrate. Necrotic lesions are associated with high numbers of eosinophils. Acanthosis is frequently encountered (28–30). Epidermotropic LCH may lead to epidermal ulceration and crusting (31). Immunohistochemical staining is essential for the diagnosis and classification of histiocytes and to rule out the mimickers. The diagnostic antibody panel for histiocytic and dendritic cell tumors may include (but is not limited to): CD1a, CD14, CD21, CD23, CD35, CD68 (less specific), CD163, CD207, fascin, podoplanin, and S100. PU.1 is a nuclear marker that distinguishes histiocytosis from histiocyte-rich tumors with an easy interpretation due to its sharp nuclear staining. Non-LCH histiocytoses display similar IHC patterns, with few exceptions (32, 33).

Specifically, Langerhans cells are recognized by their positivity for S100 protein, CD1a, and CD207 (langerin). Rare S-100 negative variants have been reported in skin-limited LCH, while CD1a and/or CD207 are required for a definitive diagnosis. In the presence of langerin, electron microscopy (revealing Birbeck granules) is no longer required to diagnose LCH. Mature LCH lesions may lack CD1a + cells and an eosinophilic infiltrate and may resemble a NLCH infiltrate (4, 8, 28, 34).

Indeterminate cell histiocytosis (ICH) is a very rare L-group histiocytosis, presenting with a generalized papular eruption, usually skin-limited. Unlike LCH, the histiocytic infiltrate is not usually accompanied by eosinophils. Immunohistochemical studies are positive for CD1a, S100, and CD68, but negative for CD207 (langerin) (4, 8, 28, 35–37).

### 3.2 RDD

RDD clinically presents with red to brown skin papules, plaques, nodules, and granulomatous lesions. RDD histiocytes are characteristically large, featuring round to oval nuclei, dispersed chromatin, prominent nucleoli, and a substantial amount of clear or foamy vacuolated cytoplasm. A notable characteristic, but not pathognomonic, of these histiocytes is emperipolesis, a process wherein the histiocytes engulf intact cells, such as neutrophils, plasma cells, and erythrocytes. Unlike phagocytosis, the cells that are engulfed retain their viability and possess the capacity to exit the histiocytes (38). Clusters of plasma cells are frequently encountered (39). The usual immunohistochemical stains in RDD are CD68, CD163, and S100 (40).

### 3.3 JXG

JXG represents the vast majority of all histiocytoses, and not LCH as previously assumed. It presents in young children as yellow-red papules or nodules, commonly on the head, neck, and upper body (41). JXGis characterized by diffuse infiltration of the superficial dermis, primarily composed of a polymorphous cell population comprising vacuolated, spindle-shaped, and xanthomatized histiocytes. The infiltration also includes a significant presence of lymphocytes, plasma cells, and eosinophils, in particular. Still, the microenvironment is not relevant to diagnosis. Touton cells (multinucleated giant cells) are not the majority but may be identified in approximately 85% of JXG cases and exhibit a distinct garland-like formation (41, 42). JXG is positive for CD163, CD68 and factor XIII-a (non-specific) (42, 43). In up to 10% of cases, JXG may involve the soft tissue, skeletal muscles, and develop ocular lesions, mimicking melanoma or neuroblastoma. Differential diagnosis with nodular-LCH may be challenging, but biopsy and immunohistochemistry are useful in this setting (44).

### 3.4 NXG

NXG typically presents as yellow to orange indurated plaques or nodules in the periorbital region. It frequently shows tendency to painful ulceration and atrophy. Although necrobiotic xanthogranuloma (NXG) is recognized as a non-XG, C group histiocytosis (4), it actually represents a paraneoplastic condition and not a primary histiocytic proliferation, as it is invariably associated with monoclonal gammopathy (44). The histological examination of NXG reveals band-shaped granulomatous inflammation characterized by foamy histiocytes, lymphocytes, and giant cells of both Touton and foreign-body types. These lesions typically extend from the mid-dermis into the subcutis, with cholesterol clefts within areas of hyaline necrobiosis and lymphoplasmacytic and mast cell infiltrates (45). The histopathological features proposed as major criteria for NXG diagnosis by Nelson et al. (46) are palisading granulomas, lymphoplasmacytic infiltrate, and zones of necrobiosis, alongside characteristic features (giant cells, cholesterol clefts) (46). Of note, although the association between NXG and paraproteinemia is well documented, the path reports do not display monoclonal plasma cells (47). NXG stains positive for: CD68, CD163, factor XIII-a, mast cells (tryptase), and polyclonal plasma cells (IgG κ chains) markers (45).

### 3.5 XD, ECD

Xanthoma disseminatum (XD) represents a cutaneous non-LCH with systemic component (4), which clinically presents with symmetrically yellow-brown confluent papules and plaques, involving the face and proximal limbs and mucous membranes (up to 30%) (44). Itdisplays Touton giant cells, foam cells, eosinophils, and positive CD68 immunostaining, with negative S-100 and CD1a (48, 49). Because of their histopathological equivalence, some authors consider XD, disseminated JXG, and Erdheim-Chester disease (ECD) clinical variants of the same disorder. XD is considered a localized ECD (50). Cutaneous manifestations of ECD consist of eyelid and periorbital xanthelasmas or other XD-like lesions; still, long-bone osteosclerosis and cardiovascular manifestations are far more frequent. Pathology characteristics for ECD are typical foamy or lipid-laden histiocytes, admixed with fibrosis, sometimes with Touton giant cells. The small histocyte aggregates have a dermal localization and do not display epidermotropism. Emperiopolesis and necrobiosis are uncommon. Immunohistochemical studies are positive for CD68, factor XIII-a and CD163 and negative for CD1a (51, 52).

### 3.6 MRH

Multicentric reticulohistiocytosis (MRH) clinically presents with papulonodular skin lesions and a symmetric and erosive polyarthritis, mimicking rheumatoid arthritis. It frequently remits spontaneously in up to 10 years (53). The histiocytes in MRH are accompanied by multinucleated giant cells and contain abundant eosinophilic granular cytoplasm (ground glass appearance), and diastase-resistant granules. Immunohistochemistry usually shows positivity for CD45, CD68, and factor XIII-a (54).

### 3.7 BRH

Multiple red to yellow-brown small papules on the head and neck of infants are typical of benign cephalic histiocytosis (BCH). The histopathological characteristics of BCH are marked by well-circumscribed histiocytic infiltrates localized primarily in the superficial to mid-dermis. The histiocytes are small, monomorphous and exhibit an abundant cytoplasm, notably devoid of cytoplasmic lipid accumulation. Accompanying this infiltrate, there is an inflammatory component consisting of lymphocytes and eosinophils. While older lesions may occasionally harbor giant cells, neither Touton cells nor foam cells are usually present within the tissue architecture. CD1a and S100 are negative, excluding LC markers, while CD68, CD163 and factor XIII-a are strongly positive (55–58).

### 3.8 SRH

Unlike the BRH, the solitary reticulohistiocytoma (SRH) is more frequent in adults and presents as a singular firm, painless yellowish nodule, usually on the head, neck, and upper trunk. The histopathological characteristics of solitary reticulohistiocytoma (SRH) are a prominent dermal infiltrate composed of mononuclear cells and multinucleated giant epithelioid histiocytes exhibiting eosinophilic, “glassy” cytoplasm. It may also include vacuolated, xanthomatous histiocytes. An inflammatory infiltrate of lymphocytes, neutrophils, and eosinophils is not uncommon. Mitotic figures are absent. SRH shows consistent positivity for CD68, factor XIII-a and CD163, while vimentin, S100, factor XIII-a, alpha-1 antitrypsin, HAM 56, and NKI/C3 (melanoma-associated antigen) often show variable positivity. Other markers, such as CD1a and CD34, are generally negative in SRH (59, 60).

Langerin stands as a confirmation marker for LCH, while non-LCH histiocytoses and ICH are generally langerin-negative. Blastic plasmacytoid dendritic cell neoplasm, myeloid dendritic cell proliferations, and dendritic cell sarcoma may rarely exhibit langerin positivity (6, 8, 28, 35, 38, 43).

## 4 Pathogenesis controversies

Histological similarities between LCH and epidermal LCs have given rise to an enduring ambivalence regarding the nature of LCH—specifically, whether it arises from the pathological activation of epidermal LC or constitutes a form of neoplastic transformation. Initially, LCH was thought to be derived from epidermal LCs based on common antigenic markers and Birbeck granules. Still, transcriptional profiling suggests that LCH is more similar to bone marrow-derived monocytes and dendritic cells than to epidermal LCs (61, 62).

Mitogen-activated protein kinase (MAPK) cascades regulate cell proliferation, survival, and differentiation. Dysregulation of these pathways contributes to neoplastic development. Among the various MAPK pathways, the extracellular signal-regulated kinase (ERK) pathway functions as a key downstream element of a well-conserved signaling cascade that is activated by three RAF serine/threonine kinases (ARAF, BRAF, CRAF). This activation process stimulates the MAPK/ERK kinase (MEK)1/2, which subsequently activates ERK 1/2. Of note, the Raf-MEK-ERK pathway serves as a primary downstream effector of the Ras small GTPase, a well-known mutated oncogene in cancer (63, 64).

Somatic mutations in the RAF-MEK-ERK pathway, especially mutations in the BRAF gene, are frequently encountered in LCH. Early activation of ERK in self-renewing progenitor or stem cells in the bone marrow can lead to a highly aggressive multi-organ disease, while activation at a later differentiation stage results in a less aggressive disease (65, 66). A mutation analysis of 38 pediatric cases of LCH found BRAF V600E mutation in approximately 37% of cases, while the ARAF mutation occurred in only 2.6% of patients. No mutations were found in the MAP2K1 and MAP3K1 genes. BRAF mutation was associated with multisystem disease, younger age (less than 2 years), and skin involvement. Additionally, the BRAF mutation was an independent predictor of LCH relapse (67).

A study involving 50 adults with LCH found a BRAF mutation in 16% of cases, with the following distribution: 30% in the skin, 11% in bone, 50% in the colon, 1% in the lung, and 33% in intracranial masses. Additionally, MAP2K1 mutations were identified in 46% of the BRAF-negative cases, with occurrence rates of 100% in lymph nodes, 50% in bones, and 25% in skin. The median age of patients with MAP2K1 mutations was 34.5 years and was comparable to the age of patients without mutations. Notably, the overall frequency of BRAF mutations in this adult cohort is significantly lower than previously reported in pediatric populations (68). Multinucleated giant cells seem to promote invasiveness in LCH via MMPs (matrix metalloproteinases) production (69). High levels of IL17A were found in LCH, but its role in LCH pathogeny remains uncertain (70).

Over 80% of ECD patients have mutations that activate the MAPK pathway. The BRAF V600E activating mutation occurs in 57–70% of cases, while MAP2K1 mutations occur in less than a third (20%) (51). A next-generation sequencing study on 21 cases of RDD did not reveal BRAF mutations, but a 33% incidence of mutually exclusive KRAS and MAP2K1 mutations, concluding on the occurrence of a MAPK pathway activation (71). MAPK1 mutation was seldom found in disseminated JXG (72), while non-disseminated forms are usually reactive to trauma or infectious stimuli (73).

Hematological malignancies are the primary systemic conditions linked to NXG, typically emerging 2–4 years after the initial appearance of skin lesions. Notably, around 80% of patients with NXG present with an associated IgM gammopathy, although rare variants of NXG can exist without monoclonal gammopathy. In cases where NXG is associated with monoclonal IgG, skin lesions may manifest up to 8 years before or even 11 years following the onset of hematological disorders. Hence, NXG may be regarded as a paraneoplastic syndrome. Still, infection with Borrelia burgdorferi plays a significant role in developing NXG and may actively trigger the disease (47, 74). Malignant histiocytoses (MH) are rare and less understood cancers and scarcely involve the skin (violaceous nodules and plaques, purpura, morbilliform rashes). Of note, MAPK mutations are more frequent in secondary MH compared to primary MH, and PTPN11 mutations are confined to primary MH (75).

MRH is characterized by a disorder of macrophage cells, specifically involving class II or non-Langerhans histiocytes, triggered by an as-yet unidentified precipitating factor. In patients with MRH, the mononuclear cells found in synovial fluid display specific macrophage phenotypes, including CD11b, CD14, and CD68. High levels of pro-inflammatory cytokines including TNF–α, IL–2, IL–6, IL–12, and IL–1β were found in these patients. The maturation of synovial macrophages into osteoclasts is mediated via the RANKL signaling pathway, which accounts for the observed responsiveness to bisphosphonates in MRH. It was shown that the ultraviolet light-induced Koebner phenomenon may play a role in the skin manifestations of MRH (76). MRH is associated with several conditions, including hyperlipidemia (up to 58% of cases), and autoimmune diseases (rheumatoid arthritis, vasculitis, Sjögren’s syndrome), affecting approximately 20% of patients. Additionally, up to 50% of MRH patients may present with a positive skin tuberculin test, but an association with tuberculosis is generally not supported. Notably, around 25% of individuals with MRH have underlying internal malignancies, prompting some researchers to classify it as a paraneoplastic syndrome (77–79).

**FIGURE 1:**
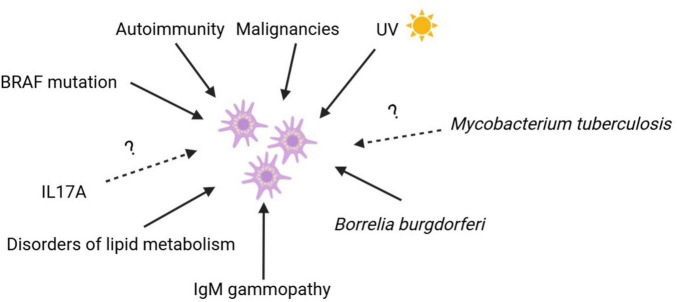
Histiocytoses pathogenesis.

Patients with XD have normal serum lipid levels, unlike other xanthomatous disorders. The exact cause of XD is unknown, but it involves the proliferation of histiocytes and lipid accumulation without hyperlipidemia. Foamy macrophages may result from increased lipid uptake, synthesis, or decreased efflux, potentially triggered by a superantigen. One hypothesis suggests that cholesterol accumulations occur secondary to the initial proliferation of histiocytes (80). Figure 1 displays the main triggers for MPS proliferation in histiocytoses.

ICH is strongly associated with myeloid neoplasms and blastic morphology represents an early histopathological predictor of an associated myeloid neoplasm (36). Of note, a systematic review of 102 cases showed that generalized eruptions and risk organ involvement in C group histiocytosis should raise a suspicion for a concomitant myeloid neoplasm. Xanthoma, xanthogranuloma and reticulohistiocytosis were most frequently encountered. More than 80% of patients with associated myeloid neoplasm had generalized lesions, and only 5.5% presented one lesion (81). It was shown that oncogene-induced senescence (OIS) might play an important role in the pathogenesis of histiocytic disorders. BRAF mutation induces a senescence program that leads to apoptosis resistance and a senescence-associated secretory phenotype, which leads to the accumulation of senescent clonal mononuclear phagocytes and the formation of LCH and ECD lesions. TNFα represents an important determinant of paracrine senescence and myeloid-restricted hematopoiesis (82–85). MAPK constitutive activation in hematopoietic stem and progenitor cells can determine an aggressive LCH (86).

## 5 Diagnostic approaches of cutaneous histiocytoses

The cutaneous presentations of histiocytosis exhibit a wide range of characteristics. Lesions with a typical rust-brown color are common in patients with LCH, MRH, RDD, and some cases of NXG. Patients diagnosed with LCH commonly present with eczematous lesions. In contrast, individuals with RDD typically exhibit granulomatous lesions (8).

Dermoscopy plays an important role in the diagnosis of JXG. A main dermoscopic feature is the “setting sun” pattern, found in both early and fully developed JXG lesions, which consists of a yellowish background accompanied by a peripheral capillary reaction (43).

**FIGURE 2:**
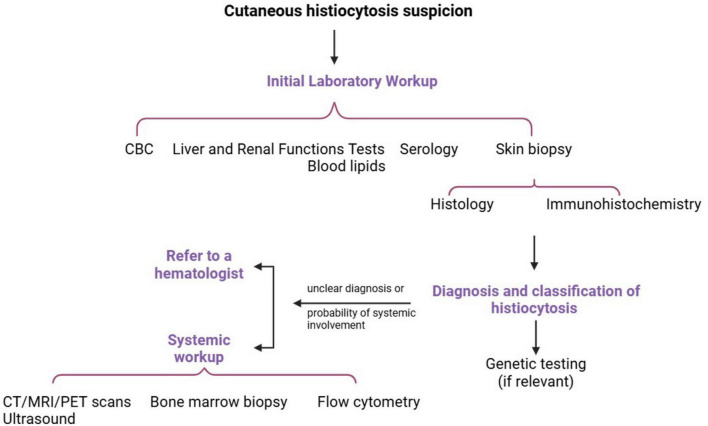
Diagnostic approach of cutaneous histiocytosis. CBC: complete blood count.

The cutaneous and general clinical evaluation will be supplemented with a standard blood workup and serology (autoimmune disease and infections screening). Until further studies, tuberculosis, and borreliosis may be excluded. The skin biopsy may be performed using either punch or excisional techniques and will be further validated through immunohistochemical analysis to establish a definitive diagnosis. When systemic involvement is clinically suspected, imaging strategies are recommended, in collaboration with a hematology consultant (Figure 2) (1, 2, 4, 8, 28, 35, 47, 74, 77).

Tables 3, 4 reveal the main diagnostic features of cutaneous histiocytoses, including clinical findings, pathology, BRAF V600 mutation status, and immunohistochemistry details.

**TABLE 3:** Skin manifestations and histology features in histiocytoses.

Histiocytosis	Pathology features	Skin manifestations
Langerhans cell Histiocytosis (LCH)	Acanthosis. proliferation of large histiocytes, pale cytoplasm, and subepidermal clustering, with grooved, ‘coffee bean’-shaped nuclei; Eosinophilic granuloma (very frequent and highly characteristic). Dermatitis lichenoides-like patterns. ± epidermotropism and epidermal ulceration.	Papules, nodules, often seborrheic dermatitis-like eruptions; ± ulceration.
Indeterminate cell Histiocytosis (ICH)	LCH-like, but lacks eosinophils.	Multiple yellow-brown papules and nodules; often widespread.
Rosai-dorfman disease (RDD)	Sheets of large histiocytes with prominent nucleoli and abundant pale cytoplasm exhibiting emperipolesis. Plasma cell clusters.	Firm, painless dermal nodules; ± overlying erythema. Granulomatous dermatitis.
Necrobiotic xanthogranuloma (NXG)	Palisading granulomas with areas of necrobiosis; cholesterol clefts; Touton cells, foam cells, giant cells.	Yellowish plaques or nodules, often periorbital; ± ulceration.
Juvenile xanthogranuloma (JXG)	Polymorphous cell population comprising vacuolated, spindle-shaped, and xanthomatized histiocytes. Plasma cells, lymphocytes, and eosinophils.	Yellow-red papules and nodules, mainly on the head and neck; self-limiting. Dermoscopy: “setting sun” pattern
Solitary reticulohistiocytoma (SRH)	Large epithelioid dermal histiocytes with abundant eosinophilic, ‘glassy’ cytoplasm. Neutrophils, lymphocytes, and eosinophils.	Solitary dermal nodule, commonly on the head, neck, or trunk.
Benign Cephalic histiocytosis (BCH)	Superficial proliferations of monomorphous histiocytes without atypia. Lymphocytes, eosinophils.	Multiple small red-brown papules, typically confined to the head and neck in infants.
Xanthoma disseminatum (XD)	Touton cells, foam cells, and eosinophils.	Widespread yellow-brown papules and nodules, often involving the mucosa.
Erdheim-Chester disease (ECD)	Foamy histiocytes surrounded by fibrosis. Lacks epidermotropism.	Xanthomatous skin lesions (especially eyelids); systemic involvement is common.
Multicentric reticulohistiocytosis (MRH)	Multinucleated giant cells with ground-glass cytoplasm and diastase-resistant granules.	Papulonodular skin lesions, typically periarticular; destructive arthritis.

LCH, Langerhans cell histiocytosis; ICH, Indeterminate cell histiocytosis; RDD, Rosai-Dorfman disease; NXG, necrobiotic xanthogranuloma; JXG, juvenile xanthogranuloma; SRH, solitary reticulohistiocytoma; BCH, benign cephalic histiocytosis; XD, xanthoma disseminatum; ECD, Erdheim-Chester disease; MRH, Multicentric reticulohistiocytosis.

**TABLE 4:** Immunohistochemistry markers and BRAF V600E mutation status of histiocytoses.

Marker	LCH	ICH	RDD	NXG	JXG	SRH	BCH	XD	ECD	MRH
CD1a	+	+	–	–	–	–	–	–	–	–
Langerin (CD207)	+	–	–	–	–	–	–	–	–	–
S100	+	+	+	–	±	–	–	–	±	–
CD68	+	+	+	+	+	+	+	+	+	+
CD163	+	+	+	+	+	+	+	+	+	+
Factor XIII-a	–	–	–	–	+	+	+	+	+	–
BRAF V600E (mutation)	±	?	–	–	–	–	–	–	±	–
Tryptase	–	–	–	+	–	–	–	–	–	–

LCH, Langerhans cell histiocytosis; ICH, Indeterminate cell histiocytosis; RDD, Rosai-Dorfman disease; NXG, necrobiotic xanthogranuloma; JXG, juvenile xanthogranuloma; SRH, solitary reticulohistiocytoma; BCH, benign cephalic histiocytosis; XD, *xanthoma disseminatum*;ECD, Erdheim-Chester disease; MRH, Multicentric reticulohistiocytosis.

## 6 Skin-directed, classic and novel therapies

The treatment of cutaneous and single-system histiocytoses is generally limited to local therapies such as surgical excision, topical agents, or observation, while multisystem histiocytoses often require systemic chemotherapy or targeted inhibitor therapies, particularly in cases with identified driver mutations or high-risk organ involvement (87–89).

### 6.1 LCH

The first-line dermatological approach to skin-limited LCH may include topical corticosteroids, calcineurin inhibitors, carmustine, and phototherapy (PUVA). Systemic thalidomide and low-dose methotrexate have beneficial effects. In cases of recalcitrant skin lesions, treatment often comprises oral corticosteroids alongside vinblastine; however, the outcomes can be unpredictable and associate with a decreased tolerance. Hence, cytarabine or cladribine is typically preferred in clinical practice. Additionally, photodynamic therapy may offer improvements for cutaneous LCH in particularly challenging instances (28, 87). Radiotherapy represents an effective and safe treatment option; even low RT doses can achieve sufficient local control (88).

### 6.2 ECD

Pegylated α-interferon has long been considered the primary treatment for patients diagnosed with ECD who exhibit bone, skin, or renal involvement. Alternative therapeutic options include anakinra, infliximab, and mTOR inhibitors such as sirolimus and everolimus. Notably, a significant 89% of patients with ECD demonstrate a positive response to allosteric MEK inhibition with cobimetinib (90, 91).

### 6.3 NXG

Immunoglobulins (IVIG), lenalidomide, and corticosteroids represent the most promising therapeutic agents for achieving disease control in NXG. IVIG demonstrates the highest responsiveness rate, followed by lenalidomide, either alone or in combination with corticosteroids, and systemic corticosteroids as a monotherapy. Additional treatments include radiotherapy, PUVA, infliximab, rituximab, and oral corticosteroids in combination with melphalan, chlorambucil, or cyclophosphamide. Furthermore, addressing any associated monoclonal gammopathy may improve the activity of skin disease, but not necessarily (92, 93). Topical treatments with steroids and nitrogen mustard are beneficial (94).

### 6.4 MRH

The treatment for MRH includes a combination of oral corticosteroids along with either cyclophosphamide or methotrexate. Intralesional steroids are beneficial. Bisphosphonates are frequently incorporated into their therapeutic approach. Few case reports have documented favorable outcomes with rituximab, tocilizumab, and anakinra (95). Among the TNF inhibitors, superior efficacy was noted for infliximab, particularly in combinations with cortico-steroids, methotrexate, or leflunomide, rather than as a monotherapy (96).

### 6.5 XD

The combination of three oral lipid-lowering molecules—peroxisome proliferator-activated receptor gamma agonist (PPAR γ), statin, and fenofibrate—was efficient in cutaneous XD. This combination inhibits the production of pro-inflammatory cytokines induced by macrophages and reduces cholesterol synthesis (97). For patients with concerns regarding cosmetic outcomes, the applications of non-ablative 1,450-nm diode lasers or pulsed dye lasers have proven to be advantageous. Systemic cyclophosphamide and cladribine are beneficial in severe cases (98, 99).

### 6.6 SRH

Cutaneous SRH exhibits a capacity for spontaneously resolving; however, surgical excision may be warranted for symptomatic relief or cosmetic purposes. The likelihood of local recurrence following incomplete surgical excision is low and is particularly infrequent following complete surgical resection (100). Non-surgical methods include PUVA and intralesional injections of triamcinolone acetonide, with promising results in combination (101).

### 6.7 BCH

BCH is self-healing in approximately 50 months and no treatments are required. However, residual post-inflammatory hyperpigmentation or atrophic scars may pose persistent and therapeutic challenges within the skin healing processes (102–104). Similarly, JXG is a generally self-healing disorder, and an observation strategy is warranted for 3–6 years. Although treatment is not required in most cases, multiple lesions are more frequently associated with extracutaneous involvement, and some authors advocate neurological and ophthalmological evaluations (43, 105). Treatment of systemic JXG consists of oral steroids, methotrexate, and vincristine (106).

**FIGURE 3:**
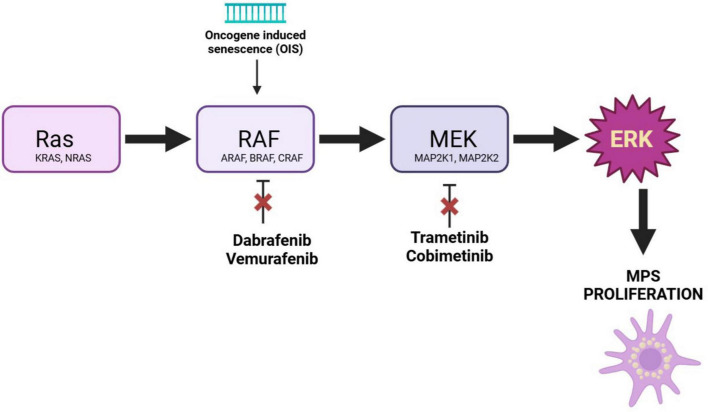
Mechanisms of action of novel therapies in multisystem histiocytoses. MPS (mononuclear phagocyte system).

BRAF inhibitors (vemurafenib and dabrafenib) in monotherapy are beneficial in adult LCH with BRAF mutation. Additionally, vemurafenib in monotherapy was efficient in infant LCH with BRAF mutation. According to Awanda et al., combined treatment with BRAF and MEK inhibitors can lead to a sustained response in LCH with BRAF mutation, as in advanced melanoma (107, 108). A recent study involving 34 patients—comprising 26 individuals with LCH, 2 with progressive systemic JXG, 2 with RDD, and 4 presenting with presumed single-site central nervous system histiocytosis—assessed the efficacy of BRAF inhibitors (Dabrafenib) and MEK inhibitors (Trametinib). These therapeutics were administered either as first-line treatment or following relapse or failure of standard chemotherapy. The findings indicated a notable responsiveness rate of 94% after a median treatment duration of 4.3 years. These therapies were determined to be both safe and effective for pediatric patients. Since Dabrafenib is effective only in patients with a BRAF mutation, the authors proposed using a BRAF inhibitor for those with BRAF V600E-associated disease and a MEK inhibitor for all other patients. They suggest that a MEK inhibitor may be beneficial regardless of mutation status (Figure 3) (109).

Treatment discontinuation and secondary adverse effects represent important issues of targeted therapies. Skin rash is the most frequent adverse event of vemurafenib in children with LCH and includes increased photosensitivity, nail abnormalities and panniculitis. Few patients may develop QT-interval prolongations, cytolysis, clonus, or sepsis. Still, response to vemurafenib persists as long as the patient remains on treatment and treatment discontinuation results in disease reactivation of most patients (84%) in the first 12 months. Hence, patients should be continuously monitored for relapse. Patients with multisystem disease have a higher relapse risk (110). Cobimetinib in ECD patients is associated with acneiform rashes, fatigue, and diarrhea, and rarely with thrombocytopenia, cytolysis, edema, and hypertension (111).

Table 5 summarizes the topical and systemic treatment options in cutaneous histiocytoses.

**TABLE 5:** Local and systemic treatments of cutaneous histiocytoses.

Histiocytosis	Local treatments	Systemic treatments
LCH (Langerhans cell histiocytosis)	Topical corticosteroids, calcineurin inhibitors, carmustine Phototherapy Photodynamic therapy Limited radiotherapy Surgical excision	Oral steroids, chemotherapy (cytarabine, cladribine, vinblastine), thalidomide, low-dose methotrexate Targeted inhibitors (BRAF, MEK inhibitors)
NXG (Necrobiotic xanthogranuloma)	Topical steroids, carmustine Local radiotherapy Phototherapy Surgical excision	Systemic corticosteroids, alkylating agents, IVIG, lenalidomide Infliximab, Rituximab
JXG (Juvenile xanthogranuloma)	Observation, surgical excision	None typically required
SRH (Solitary reticulohistiocytoma)	Surgical excision Phototherapy Intralesional corticosteroids	None typically required
BCH (Benign cephalic histiocytosis)	Observation (self-limiting)	None typically required
XD (Xanthoma disseminatum)	Diode lasers Pulsed dye laser Surgical excision of isolated lesions	Combination of oral lipid-lowering molecules—peroxisome proliferator-activated receptor gamma agonist (PPAR-γ), statin, and fenofibrate. Systemic corticosteroids, cyclophosphamide, cladribine.
ECD (Erdheim-Chester disease)	Surgical debulking if localized, otherwise limited role	Targeted inhibitors (BRAF, MEK inhibitors), interferon-alpha, chemotherapy. Alternatives: anakinra, infliximab, mTOR inhibitors.
MRH (Multicentric reticulohistiocytosis)	Intralesional corticosteroids	Oral steroids, cyclophosphamide, methotrexate. Bisphosphonates. Infliximab (in combination)

## 7 Conclusion

The recent classifications of histiocytosis have significantly clarified their cellular origins, enabling the adoption of more effective diagnostic and management techniques. The skin biopsy represents an essential step for accurately diagnosing cutaneous histiocytosis. With advancements in histopathological and immunohistochemical studies, healthcare professionals can now achieve definitive diagnoses, moving away from ambiguous terms like “non-Langerhans cell histiocytosis.” CD1a and langerin stand as important criteria for LCH diagnosis and langerin-negative cases in indeterminate cell histiocytosis are of particular interest to the clinician.

Identifying BRAF mutations has opened up exciting opportunities for targeted therapies, such as Dabrafenib, that can greatly improve patient outcomes. For those without BRAF mutations, MEK inhibitors (Trametinib) present another promising treatment avenue. Furthermore, therapies including IVIG, immunosuppressants, mTOR inhibitors, and TNF inhibitors are emerging as viable options, shifting away from classical chemotherapy for multisystem disease. Topical treatments, including corticosteroids and alkylating agents, as well as steroid injections, phototherapy, and laser-based therapies, have demonstrated significant efficacy in managing skin lesions, providing measurable therapeutic benefits. Caution should be exercised with phototherapy, as UV radiation may potentially exacerbate lesions. An observation strategy is recommended for BCH and JXG.

Despite the challenges posed by the relatively small number of cases, which has impacted the creation of universally accepted treatment guidelines and large-scale studies, ongoing research and collaboration in this field may hold great potential for advancing care and improving patient prognosis.
